# Chest X-ray Reporting: A Comparative Study of Specialist Nurses and Trainee Doctors’ Knowledge in the Biologic Prescription Service

**DOI:** 10.7759/cureus.48801

**Published:** 2023-11-14

**Authors:** Awin Mohammed Murad, Fatima Farman, Kehinde O Sunmboye

**Affiliations:** 1 General Internal Medicine, University Hospitals of Leicester NHS Trust, Leicester, GBR; 2 Health Sciences, University of Leicester, Leicester, GBR; 3 Rheumatology, University Hospitals of Leicester NHS Trust, Leicester, GBR

**Keywords:** training for specialist nurses, workforce crisis, clinical nurse specialist, pre-biologic workup, chest x-ray interpretation

## Abstract

With all the challenges facing the NHS at the current time, specialist nurses are fundamental and an important part of an ever-expanding NHS workforce. Furthermore, specialist nurses now possess more diversity and a wide range of advanced skills.

In the field of rheumatology in most NHS hospitals, specialist nurses play a key role in biologic services to ensure that patients are promptly started on biological therapy to control their disease. An important element of this workup is the ability to comment on an unreported chest radiograph to facilitate a biological prescription. Some studies have shown that there is limited expertise among non-doctors with the required skills to review unreported chest X-rays confidently.

The authors of this paper sought to explore whether this is the case among specialist nurses involved in the biologic prescription service among other clinicians in the same service. An online questionnaire was designed by the authors, which included seven questions and responses collected on a 5-point Likert scale. Trainee doctors, non-trainee grade doctors, and specialist nurses who were involved in the biologic prescribing team from Rheumatology, Dermatology, and Gastroenterology were invited.

A total of 56 responses were obtained and analyzed. Descriptive and inferential statistics were obtained from the data. To determine if there was a statistical difference between the responses of trainee doctors and specialist nurses, the Kruskal-Wallis statistical test was used, and a post hoc test using the Dunn-Bonferroni test was used to analyze any statistically significant results.

Regarding chest X-ray interpretation prior to starting biological treatment, only 8% of specialist nurses reported being confident, whereas 63% of trainees reported being confident. The Kruskal-Wallis test revealed a significant difference between specialist nurses' and doctors’ confidence in interpreting unreported chest radiographs. The P-value is 0.001; thus, with available data, the null hypothesis is rejected. A Dunn-Bonferroni test (post hoc test) showed that, based on the available data, it can be assumed that the two groups had different levels of confidence between Specialist Nurses and trainee doctors.

Chest X-ray interpretation skills are vital for specialist nurses in the context of biological therapy prescriptions. Therefore, we recommend access to resources, ongoing formal training, and educational sessions to help specialist nurses maintain their advanced skill sets and broaden their scope of practice to those without the required expertise.

## Introduction

With the increasing demand for NHS services, specialist nurses are becoming increasingly involved in inpatient management decisions, education, and follow-up reviews. A specialist nurse is a registered nurse with a master’s or doctoral degree, specializing in a specific field of clinical medicine with advanced skills [1]. They have been an integral part of the changing culture of the NHS as they have been more empowered through skills training and development to perform more specialized tasks [2]

In the United Kingdom, specialist nurses are taking on more advanced roles, such as administering intra-articular injections, monitoring disease-modifying treatment, prescribing drugs, and managing comorbidities through nurse-led clinics, especially in the field of rheumatology [3].

According to a Royal College of Nursing factsheet, specialist nurses perform routine patient follow-ups, allowing consultants to see new patients [4]. This has saved £175,168 per annum per nurse whole time equivalent (WTE). Furthermore, centers having access to specialist nurse-led phone consultations have deferred 60% of patients from requesting a general practitioner (GP) appointment [5].

Studies have revealed that appropriately using the role of specialist nurses has contributed to reduced patient morbidity and improved quality of care [6]. A recent rheumatology study concluded that, in the next several decades, there will be a shortage in the rheumatology workforce. Therefore, mid-level medical professionals, such as nurse practitioners and physician assistants should play an extended role [7].

Specialist nurses are increasingly included within specialist teams. Within the rheumatology departments of most hospitals, specialist nurses play a key role in the biologics service to ensure that patients are started promptly on biologic therapy to treat their diseases [8]. These specialist nurses are usually involved in reviewing chest radiographs as part of processing the biologic therapy medication required by patients whose treatment needs to be escalated to control the disease [9]. Nurses with adequate formal training in chest X-ray interpretation possess the assessment skills required to carry out the tasks they are given [10]. There is however a lack of formal training for nurses in basic X-ray interpretation which may impact negatively on patients if this skill is required in health care delivery [11,12].

One of the challenging aspects of being a specialist nurse working as part of the biologics team in a rheumatology department is the lack of formal training for all nurses who perform this role. This tends to negatively impact their confidence to comment on an unreported chest radiograph as part of a pre-biologic workup. Therefore, this study assessed the confidence of rheumatology specialist nurses in commenting on chest X-rays without a formal report compared to other doctor grades ranging from first-year doctors to experienced rheumatology specialist registrars in training or non-training trust grade doctors involved in the biologic prescribing team.

## Materials and methods

Study design and setting 

This cross-sectional study was conducted at Leicester Royal Infirmary, University Hospitals of Leicester NHS Trust, a busy university teaching hospital in the East Midlands of the United Kingdom between May 2023 and August 2023. The hospital serves as the primary accident and emergency service provider for its area in the East Midlands, encompassing approximately 1000 beds. The Leicester Central Research Committee (EM-Leicester Central REC) of the hospital issued approval REC:2023-0001.

Participants

Participants were trainee and non-trainee-grade doctors and specialist nurses who were involved with the biologic prescribing team in the East Midlands region of the UK. Clinicians from Rheumatology, Dermatology, and Gastroenterology specialties were invited to participate in this study. All participants provided informed consent, and no refusal was encountered. None of the responses were excluded. Data were collected through an anonymous online questionnaire, which took approximately five minutes to complete. The questionnaire was distributed to participants via email. 

Eligibility criteria

Inclusion Criteria

All trainee and non-trainee grade doctors and prescribing specialist nurses involved in biological prescriptions across Rheumatology, Dermatology, and Gastroenterology specialties. 

Exclusion Criteria

Clinicians not involved in biologic prescribing services across Rheumatology, Dermatology, and Gastroenterology specialties. 

Data source and collection

An anonymous online questionnaire was distributed to all the participants. The questionnaire consisted of seven questions, each with a 5-point Likert scale response format, to assess the following aspects: (1) familiarity with pre-biologic workup for patients, (2) confidence in identifying contraindications to biologic treatment from history, (3) confidence in interpreting chest X-rays before starting biologic therapy in patients, (4) confidence in commenting on unreported chest X-rays to facilitate prescriptions of biologic therapy, and (5) the need for more training in chest X-rays (if any required).

Outcomes

The primary outcome of the study was the confidence of participants in interpreting chest X-rays in patients prior to commencing biologic therapy. This was assessed using a 5-point Likert scale (1 = strongly disagree, 5 = strongly agree).

The secondary outcomes were (1) the perceived level of expertise in chest X-ray interpretation and (2) the perceived level of training in chest X-ray interpretation required (if any). 

Statistical software 

All statistical analyses were performed using DATAtab® (DATAtab e.U., Graz, Austria), an online statistical software analysis tool. 

Statistical methods 

The Kruskal-Wallis test was used to compare the confidence of trainee and non-trainee-grade doctors and specialist nurses in interpreting chest X-rays. A post-hoc Dunn-Bonferroni test was used to analyze any statistically significant results. 

Bias

The following potential biases were mitigated in this study: (1) selection bias-all clinicians and specialist nurses involved in biological prescriptions were invited to participate in the study, (2) non-response bias-every effort was made to maximize the response rate, including sending multiple reminders to the participants, and (3) measurement bias**-**the questionnaire was pilot-tested to ensure that it was clear and easy to understand. 

Sensitivity analysis

A sensitivity analysis was performed to assess the impact of excluding participants with missing data. The results of the sensitivity analysis were consistent with the main results of the study. 

## Results

A total of 56 healthcare professionals completed the questionnaire. There were 1 foundation year 1 (F1)/F2, 5 core trainee level 1 (CT1)/CT2, 33 higher specialty trainees, 13 rheumatology specialist nurses, and 4 trust-grade doctors (Table 1). All correspondences were divided into two groups to compare confidence between trainees (F1/F2, CT1/CT2, trust-grade doctors, and higher specialty trainees) and rheumatology-specialist nurses.

**Table 1:** Grade of clinicians CT1/CT2: core trainee level 1/core trainee level 2 F1/F2: foundation year 1/foundation year 2

Grade of clinician	Number	Percentage
Higher specialty trainee	33	59%
CT1/CT2	5	9%
F1/F2	1	2%
Trust grade doctor	4	7%
Rheumatology nurse specialist	13	23%
Total	56	100%

**Figure 1:**
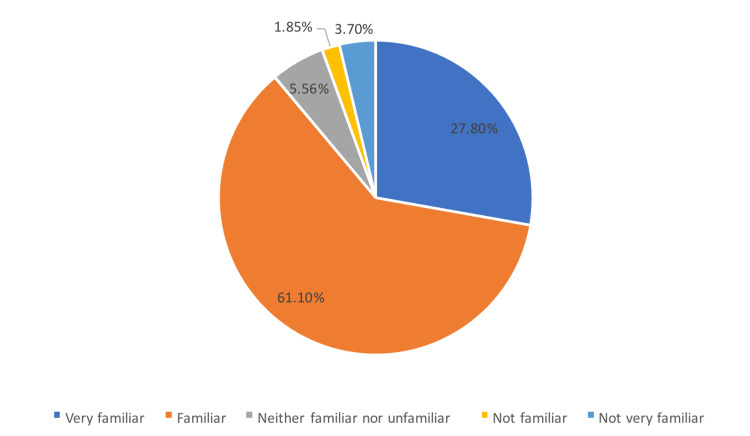
Familiarity with pre-biological workup for patients with rheumatic disease needing biologics

When assessing familiarity with pre-biologic workup for patients with rheumatics disease requiring biologics, overall, 61.1% of the respondents reported that they were familiar and 27.8% very familiar (Figure 1). Meanwhile, only 3.7% reported that they were not very familiar, and 1.85% not familiar.

**Figure 2:**
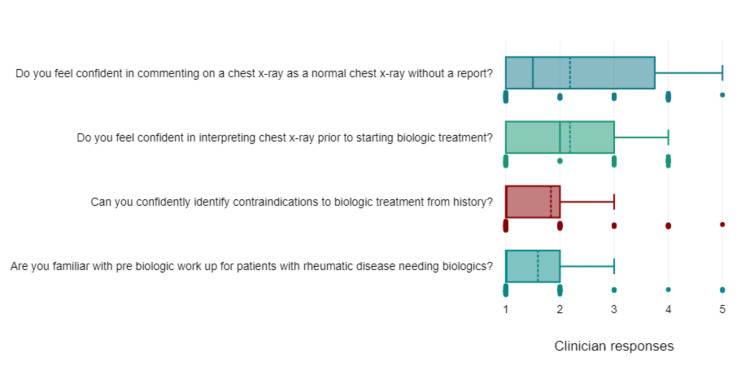
Box plot showing clinician responses Key Likert scale responses from 1 to 5: 1, Not confident; 2, Not very confident; 3, Neither not confident nor confident; 4, confident, 5, very confident

Among respondents, 93% of specialist nurses and 86% of trainees were familiar with pre-biologic workups for patients with rheumatic disease, while 70% of specialist nurses and 60% of trainees reported being confident in identifying contraindications to biologics from history. Regarding chest X-ray interpretation prior to starting biologic treatment, only 8% of specialist nurses reported being confident, meanwhile, 65% of trainees reported being confident (Figure 2).

Finally, when asked if more training was required to interpret chest X-rays, 85% of the specialist nurses answered yes, and 63% of the trainees gave the same answer. When specialist nurses were asked what level of training was required, 62% reported full training, 30% some training, and 8% no training.

To determine whether there was a statistical difference between the responses of nurses and doctors. Kruskal-Wallis statistical tests were used, which is a non-parametric hypothesis test best suited for the alternative hypotheses in this study. A post hoc test using the Dunn-Bonferroni test was then used to analyze any statistically significant results to determine the actual difference between groups.

The Kruskal-Wallis test shows (Table 2) that there was a significant difference between the categories of the independent variable with respect to the dependent variable, "Do you feel confident in interpreting a chest X-ray prior to starting biologic treatment?" The p is 0.001. Thus, the null hypothesis was rejected using available data. The Kruskal-Wallis test revealed significant differences. Dunn-Bonferroni post hoc test was used to compare the groups in pairs to determine which groups were significantly different. The Dunn-Bonferroni test showed that the pairwise group comparison of higher specialty trainee-rheumatology nurse specialists had an adjusted p-value of less than 0.05, and thus, based on the available data, it can be assumed that the two groups are significantly different; hence, the two groups are significantly different from each other in the level of confidence in this area (Table 3).

**Table 2:** Kruskal-Wallis test to compare confidence in chest X-ray reporting prior to biologics between independent and dependent variable

	Chi^2^	df	p
Do you feel confident in interpreting chest X-ray prior to starting biologic treatment?	20	4	0.001

**Table 3:** Dunn-Bonferroni test (post hoc test) to compare which groups exhibited statistically significant differences regarding confidence in chest X-ray interpretation prior to biologics Adj. p: Values adjusted with Bonferroni correction CT1/CT2: core trainee level 1/core trainee level 2 F1/F2: foundation year 1/foundation year 2

Groups	Test Statistic	Std. Error	Std. Test Statistic	p	Adj. p
Higher specialty trainee - CT1/CT2	-13	7	-2	0.06	0.603
Higher specialty trainee - F1/F2	9	15	1	0.562	1
Higher specialty trainee - Trust grade	-7	8	-1	0.391	1
Higher specialty trainee - Rheumatology nurse specialist	-21	5	-4	<0.001	<0.001
CT1/CT2 - F1/F2	22	16	1	0.174	1
CT1/CT2 - Trust grade	7	10	1	0.505	1
CT1/CT2 - Rheumatology nurse specialist	-8	8	-1	0.304	1
F1/F2 - Trust grade	-15	16	-1	0.351	1
F1/F2 - Rheumatology nurse specialist	-30	15	-2	0.05	0.503
Trust grade - Rheumatology nurse specialist	-15	9	-2	0.086	0.862

The Kruskal-Wallis test shows (Table 4) that there is a significant difference between the categories of the independent variable with respect to the dependent variable, "Do you feel confident in commenting on a chest X-ray as a normal chest X-ray without a report?" The p is <0.001. Thus, the null hypothesis was rejected using the available data. Dunn-Bonferroni test (post hoc test) was used to compare the groups in pairs to determine which was significantly different. The Dunn-Bonferroni test revealed that the pairwise group comparisons of higher specialty trainee-rheumatology nurse specialist, CT1/CT2-rheumatology nurse specialist, and trust grade-rheumatology nurse specialist have an adjusted p-value less than 0.05; thus, based on the available data, it can be assumed that these groups are significantly different in these pairs (Table 5).

**Table 4:** Kruskal-Wallis test to compare confidence on commenting on a chest X-ray as a normal chest X-ray without a report between independent and dependent variable

	Chi^2^	df	p
Do you feel confident in commenting on a chest X-ray as a normal chest X-ray without a report?	21	4	<0.001

**Table 5:** Dunn-Bonferroni test (post hoc test) to compare which groups exhibited statistically significant differences regarding confidence in commenting on a chest X-ray as a normal chest X-ray without a report Adj. p: Values adjusted with Bonferroni correction CT1/CT2: core trainee level 1/core trainee level 2 F1/F2: foundation year 1/foundation year 2

Groups	Test Statistic	Std. Error	Std. Test Statistic	p	Adj. p
Higher specialty trainee - CT1/CT2	5	7	1	0.445	1
Higher specialty trainee - F1/F2	-13	15	-1	0.392	1
Higher specialty trainee - Trust grade	4	8	1	0.585	1
Higher specialty trainee - Rheumatology nurse specialist	-21	5	-4	<0.001	<0.001
CT1/CT2 - F1/F2	-18	16	-1	0.259	1
CT1/CT2 - Trust grade	-1	10	0	0.908	1
CT1/CT2 - Rheumatology nurse specialist	-26	8	-3	0.001	0.009
F1/F2 - Trust grade	17	16	1	0.3	1
F1/F2 - Rheumatology nurse specialist	-8	15	-1	0.593	1
Trust grade - Rheumatology nurse specialist	-25	9	-3	0.003	.033

There was numerical significance among the clinicians that more chest X-ray training was required but there was no statistical significance between the groups (Table 6, 7).

**Table 6:** Kruskal-Wallis test for the need for more training in chest X-ray reporting between dependent and independent variable

	Chi^2^	df	P
Do you feel you need more training in interpreting chest X-ray?	3	4	0.544

**Table 7:** need for more training in chest X-ray reporting between different grades CT1/CT2: core trainee level 1/core trainee level 2 F1/F2: foundation year 1/foundation year 2

		Do you feel you need more training in interpreting chest X-ray?	
		No	Yes
Grade of Clinician	Higher specialty trainee(n=33)	13	20
	CT1/CT2(n=5)	1	4
	F1/F2(n=1)	0	1
	Trust grade(n=4)	2	2
	Rheumatology nurse specialist(n=11)	2	9
	Total(n=56)	18	38

## Discussion

One of the main challenges facing rheumatology is the gap between the demand for rheumatologists in various regions of the UK and the current workforce shortage. Specialist nurses could bridge this gap by providing high-quality care [13]. In the United States, a study compared care provided by nurse practitioners and physician assistants versus sub-specialist physicians only and concluded that nurse practitioners can efficiently diagnose rheumatoid arthritis, make adjustments in medications, and a utilize treat-to-target strategy [7]. With the knowledge gap identified in our study, formal training will help improve this issue and allow specialist nurses to help bridge this gap in most specialties in the UK. 

**Figure 3:**
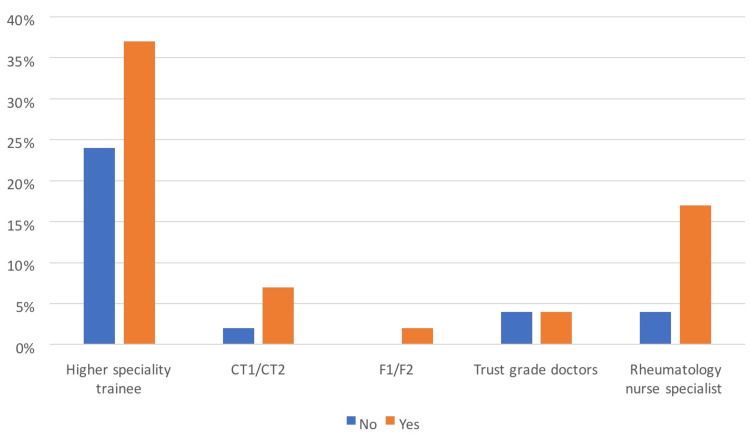
Comparing the need for more training in interpreting chest X-rays between different grades of clinicians CT1/CT2: core trainee level 1/core trainee level 2 F1/F2: foundation year 1/foundation year 2

The specialist nurses who participated in the study have identified that formal training will help them confidently comment on chest X-rays to exclude contraindications to biologic therapy thereby facilitating biologic therapy prescribing (Figure 3). This is consistent with one of the reasons identified in a study that showed that nurses do not have any formal training in X-ray interpretation, as there is no unified approach to level the variation in educational methods and requirements [11].

While our data also revealed that trainees, even at the third-year level of training, are more confident in interpreting chest X-rays than rheumatology nurses (Table 3), various studies have shown that through formal training, specialist nurses can acquire complex skills within a defined specialty [14]. Overton-Brown and Anthony assumed that with increased experience, comes a higher level of accuracy [15]. Another study in the UK assessed the diagnostic accuracy and certainty of chest X-ray interpretation in the medical division. The study concluded that consultants and higher specialty trainees scored the highest average certainty levels, junior trainees and other healthcare professionals felt least certain, and their diagnoses were more likely to be wrong [16].

Nevertheless, lifelong learning and nurses’ continuing professional development will help bolster skill acquisition [14].

A study was conducted in London by Aitkenhead and Lee to assess the accuracy of pediatric limb radiograph interpretation by nurse practitioners in a single center, and pediatric limb radiographs interpreted by nurse practitioners were compared against the gold standard of consultant radiologists [17]. The study concluded that the sensitivity of limb radiographic interpretation among nurses with training was 92%, with a specificity of 78%. The study also explained that nurse practitioners had a one-day course that provided a structured approach for interpreting radiographs requested in the emergency department. However, the study suggested that the specialist nurses still relied on clinical experience in interpreting radiographs rather than through formal training although they acknowledged its usefulness. 

There is no doubt that specialist nurses play an essential role in the current medical workforce, as they provide patient education, monitor a patient’s condition, provide psychological support, and are involved in research. Therefore, they require a high level of training and education to provide a high level of care. 

One limitation of our study was the small sample size, as it was difficult to obtain responses from all trainees and specialist nurses in the region involved in the biologics team. Despite this limitation, the findings are useful in ensuring that specialist nurses have access to formal training to allow them to do more to enhance patient care and health care delivery.

## Conclusions

Since there is an increasing demand for NHS services, many specialist nurses have the opportunity to expand their scope of practice, and chest X-ray interpretation skill acquisition can be considered an important skill that specialist nurses can acquire, which will help facilitate efficient delivery of care not only within the context of biologic therapy prescribing but also within the wider health economy. We therefore recommend that access to formal training and teaching sessions should be the norm for specialist nurses without barriers that prevent those willing to engage in this training to broaden their scope of practice.
